# Challenges in Hemodialysis: An Analytic Study of Nurses’ Cannulation Failures

**DOI:** 10.3390/healthcare14081077

**Published:** 2026-04-17

**Authors:** Fatmah Ahmed Alamoudi, Mahmoud Abdel Hameed Shahin, Maryam Abdullah Bayahya, Shouq Mubarak Al Zuabi, Rasha Essam Bakhurji, Wadha Anbar Aldarbi, Hanan Alfahd

**Affiliations:** 1 Nursing Department, Prince Sultan Military College of Health Sciences, Dhahran 34313, Saudi Arabia; fatimaamodi@psmchs.edu.sa; 2 Hemodialysis Unit, King Fahad Military Medical Complex, Dhahran 34313, Saudi Arabia; mariamslae2024@gmail.com (M.A.B.); shouq905@gmail.com (S.M.A.Z.); wadhaaldarbi@gmail.com (W.A.A.); 3 Health Informatics, Nursing Administration, King Fahad Military Medical Complex, Dhahran 34313, Saudi Arabia; rasha5005@yahoo.com; 4 Nursing Department, King Fahad Military Medical Complex, Dhahran 34313, Saudi Arabia; hanan.fahd@kfmmc.med.sa

**Keywords:** chronic kidney disease, renal failure, hemodialysis, Saudi Arabia, nurses cannulation failure

## Abstract

**Background/Objectives**: Nurses and dialysis technicians are primarily responsible for cannulation in in-center and satellite dialysis units. Despite being a core component of hemodialysis care, existing clinical guidelines offer limited standardization, resulting in practice variability across facilities. Therefore, clinical expertise and adherence to consistent standards are essential to ensure safe and effective vascular access management. The study aimed to investigate the variables related to patients and nurses that contribute to unsuccessful vascular access cannulations, as well as the actions taken in response to cannulation failure, in a tertiary dialysis center in the Eastern Region of Saudi Arabia. **Methods**: This retrospective analytic study reviewed the records of 228 adult hemodialysis patients at King Fahad Military Medical Complex from 2020 to 2024, analyzing demographic, clinical, vascular access, and nursing variables associated with cannulation failure using descriptive statistics, the chi-square test, and t-tests. Ethical approval was obtained, and data were de-identified and manually extracted from nursing and dialysis documentation. **Results**: Most patients had hypertension and diabetes, with significant comorbidity burdens. Infiltration (61%) and clot formation (30.7%) were the primary complications of cannulation failure. Significant associations emerged with recurrent stroke and peripheral vascular disease, but not with nurse or patient demographics, suggesting vascular factors outweigh staff variables in cannulation risk. Cannulation failures were most common in patients with vascular comorbidities, while staff experience and education had no significant impact. **Conclusions**: Recommendations include implementing tailored protocols, providing ongoing nurse education, conducting systematic vascular assessments, and holding regular team reviews to enhance access outcomes and patient safety.

## 1. Introduction

Hemodialysis is a vital treatment for people with end-stage renal failure, allowing the removal of waste products, toxins, and excess fluids from the blood. To initiate this treatment, a reliable vascular access point is required, which delivers a secure and efficient conduit for repeated blood circulation between the patient and the dialysis machine [1]. While central venous catheters (CVCs) are commonly used for immediate access, particularly in urgent or early-stage cases, their prolonged use is associated with increased risks of infection, thrombosis, and cardiovascular complications [2,3]. Therefore, Arteriovenous fistulas (AVFs) and arteriovenous grafts (AVGs) serve as the primary forms of permanent vascular access for patients undergoing hemodialysis, with AVFs generally selected due to their lower risk of infection, greater long-term patency, and association with improved health-related quality of life [4,5]. Nevertheless, the selection and functional outcome of vascular access are often influenced by patient-specific factors, including aging and the presence of comorbidities [4].

Although vascular access plays a crucial role in hemodialysis, emerging evidence suggests that related complications present significant challenges to the successful delivery of treatment [6,7]. In clinical practice, repeated cannulation failure can compromise the integrity of the access site and give rise to various complications, including infiltration, hematoma formation, thrombosis, and venous stenosis [7,8]. Over time, these complications may result in permanent loss of access and significantly disrupt the efficiency and continuity of dialysis treatment [7]. In addition, recurring cannulation failures can cause significant pain and may lead to heightened anxiety, fear, and psychological distress for the patient, which ultimately affects the overall quality of life [9]. In Saudi Arabia, the prevalence of end-stage renal disease (ESRD) is increasing, with 28,256 cases reported by the Saudi Center for Organ Transplantation (SCOT) in 2019 [10]. As most of these patients require ongoing hemodialysis, it is essential for nurses working in dialysis units to possess comprehensive knowledge of vascular access and its potential early and late complications [11].

Nurses and dialysis technicians assume primary responsibility for cannulation procedures in both in-center and satellite dialysis units [9]. Although cannulation is fundamental to hemodialysis care, current clinical guidelines offer limited detail on standardized techniques, resulting in considerable variation across facilities [12,13]. Cannulation of vascular access is a multi-step procedure that may involve ultrasound guidance for needle placement, the application of a tourniquet, and the use of local anesthesia [9]. Commonly used methods, such as the rope ladder, area, and buttonhole techniques, differ in terms of needle site rotation and insertion precision [12,13]. Nurses make essential decisions during cannulation, such as choosing the needle’s direction, size, and insertion approach, as these decisions can significantly impact the vascular access function and patients’ outcomes [9]. Due to these variations in practice, clinical experience and consistent standards are crucial for ensuring safe and effective vascular access management.

Most existing research on vascular access has focused on long-term outcomes, particularly the time from access creation to definitive failure [14,15,16]. While the literature offers limited insight into the procedural success of cannulation, notable investigations by Parisotto et al. [12] identified several technical factors associated with unsuccessful cannulation, including the use of an arteriovenous fistula (AVF) instead of arteriovenous graft (AVG), a shortened cannulation path, use of back-eye needles, rope-ladder technique, initial placement of the venous needle, arterial needle rotation, and the use of 16–17-gauge needles [15]. However, these studies primarily explored access-related and procedural variables while largely overlooking the contributions of patient characteristics and nurse-related factors. To date, limited research has examined the influence of patients’ or nurses’ factors on cannulation failure, particularly in underrepresented regions such as Saudi Arabia. Moreover, little is known about how nurses respond to cannulation failures in real-world clinical practice.

Although several studies have examined vascular access failure and related complications in hemodialysis patients [5,7,17,18,19], most have focused on long-term patency or access survival rather than on day-to-day cannulation success at the unit level. Existing research from Saudi Arabia and the broader Middle East is particularly limited, with few studies assessing patient-, access-, and staff-related predictors of unsuccessful cannulation in routine practice. Furthermore, there is a lack of a comprehensive conceptual framework integrating clinical, anatomical, and procedural factors that may jointly contribute to cannulation failure, especially in high-volume tertiary centers. Therefore, this retrospective analytic study was conducted to identify factors associated with unsuccessful vascular access cannulation among hemodialysis patients and the actions taken in response to cannulation failure in a tertiary center in the Eastern Region of Saudi Arabia, addressing these methodological and theoretical gaps.

### Significance of the Study

There is a scarcity of data regarding the variables that affect effective cannulation [9,20], especially in Saudi Arabia. Moreover, there is an absence of clinical guidelines and recommendations on cannulation techniques, as well as evidence to support best practices [9,12,13]. Further research is necessary to identify the many patient and vascular access characteristics, as well as patient and nurse-related variables, that contribute to miscannulation and access-related problems. This study aims to investigate the variables related to patients and nurses that contribute to unsuccessful vascular access cannulations, as well as the actions taken in response to cannulation failure, in a tertiary dialysis center in the Eastern Region of Saudi Arabia.

## 2. Materials and Methods

### 2.1. Design

The study employed a single-centered retrospective cohort study design to identify factors associated with unsuccessful vascular access (VA) cannulation in 228 hemodialysis patients. All adult hemodialysis patients treated at a tertiary center in the Eastern Region of Saudi Arabia between 1 January 2020, and 31 December 2024, who had at least one unsuccessful VA cannulation during the study period were eligible. Data were obtained from electronic medical records and dialysis unit logs.

### 2.2. Data Source

The de-identified data were obtained from the records of the Hemodialysis Unit at King Fahad Military Medical Complex (KFMMC). Dialysis documentation and nursing records were used as the primary data sources for this study. Data were extracted manually by the research team under the supervision of the principal investigator and were approved by the Institutional Review Board at KFMMC.

### 2.3. Study Population and Setting

Using de-identified records from the Hemodialysis Unit at KFMMC, the eligibility criteria of this retrospective study included only adult patients who: (1) received chronic hemodialysis treatment at KFMMC between 1 January 2020, and 31 December 2024; (2) were 18 years of age or older; and (3) had at least one documented cannulation failure involving an AVF or AVG. Patients undergoing dialysis through CVCs rather than AVF or AVG were excluded from the study. The final cohort comprised 228 patients who met the inclusion criteria. We analyzed only the first failure per patient without repetition as indicated by the patient’s hospital record number.

### 2.4. Study Variables and Measurement

Demographic variables included age, sex (female vs. male), and comorbidities such as diabetes mellitus, hypertension, stroke, and peripheral vascular disease. Vascular access variables included the type of access (AVF vs. AVG). During data collection, no restrictions were applied regarding the anatomical location of AVF or AVG. Access sites included the forearm and upper arm, specifically the cephalic, basilic, and saphenous veins of the upper extremities (radiocephalic, brachiocephalic, and brachiobasilic). No AVF or AVG sites were identified in the lower extremities.

Various vein sizes were cannulated; however, all were mature veins, as determined in accordance with the hospital policy on AVF assessment, which includes the “Rule of 6s,” ultrasound evaluation when indicated, and physical examination before each cannulation. The Rule of 6s is a clinical guideline used to determine whether an arteriovenous fistula (AVF) has matured sufficiently for successful hemodialysis cannulation, typically evaluated about 6 weeks after creation. A mature fistula is characterized by adequate vein diameter, appropriate depth from the skin surface, and sufficient blood flow.

According to the commonly accepted Rule of 6s criteria, a mature fistula should have a blood flow greater than 600 mL/min, a vein diameter of at least 6 mm (0.6 cm), a vein depth less than 6 mm (no more than 0.6 cm below the skin surface), and at least 6 cm of usable straight vein for cannulation. When these criteria are met, the fistula is more likely to support successful two-needle dialysis. It is also worth noting that, in accordance with hospital policies and procedures, cannulation of an aneurysmal fistula is prohibited in nursing practice.

The AV fistula blood flow during dialysis is typically set at 300 mL/min, in accordance with hospital policies and procedures. The B. Braun IQ dialysis machine is used, along with the B. Braun fistula needles. Needle gauges of 15, 16, and 17 are available in two lengths: 20 mm and 25 mm. During cannulation, the distance between the two needles is usually at least 3 cm, and the rope ladder technique is the standard vascular access method used in the hospital.

Patients had normal skin; none had the abnormally thin or thick skin often seen in individuals treated with corticosteroids or those with long-standing type I diabetes. Usually, if the next session patient has a failure of attempt after proper assessment, an ultrasound is performed in the unit to determine the reason. If advanced investigation is needed, a venogram is booked for the patient in radiology, and the necessary intervention is performed there. Sometimes, upon performing the ultrasound in the unit, the vascular surgeon can determine the issue and admit the patient for surgery. The duration of dialysis via the fistula from the first session to the first failure varied depending on complications that developed after cannulation and connection to the dialysis machine, such as stenosis, ischemic symptoms in the hand (steal syndrome), and thrombosis.

The primary outcome was unsuccessful cannulation, defined as the inability to complete a dialysis session due to access-related complications. Documented consequences of cannulation failure were recorded and categorized as infiltration, clot formation, immature fistula, thrombosis, or venous stenosis. In addition, actions taken in response to cannulation failure were reviewed and grouped into re-needling, catheter insertion, or discontinuation of dialysis.

Nurse-related variables included level of education (diploma vs. bachelor’s degree or higher) and years of experience (less than 5 years vs. 5 years or more). All variables were collected from dialysis documentation and nursing records during the study period.

### 2.5. Statistical Analysis

Descriptive statistics were used to summarize patient demographics, vascular access characteristics, nurse-related variables, and the outcomes of cannulation failure. There was no missing data for the variables included in the analysis. Continuous variables were presented as means with standard deviations, while categorical variables were expressed as frequencies and percentages. Chi-square tests were used to examine the associations between cannulation failure outcomes and categorical variables, including comorbidities, type of vascular access, and nurse experience. Where appropriate, Fisher’s exact test was used for expected cell counts below five. To explore the relationship between patient-, access-, and nurse-related variables and specific types of cannulation failure, cross-tabulations were performed with corresponding chi-square analyses. We examined associations using stratified analyses by vascular access type and key comorbidities to reduce confounding and bias. Statistical significance was set at a *p*-value of <0.05. All analyses were conducted using the Statistical Package for the Social Sciences (SPSS), version 30 (IBM Corp, Armonk, NY, USA).

### 2.6. Ethical Considerations

Using de-identified records from the Hemodialysis Unit at KFMMC, this retrospective study was approved by the KFMMC Institutional Review Board (IRB#AFHER-IRB-2024-045). All ethical considerations of scientific research were strictly adhered to in accordance with the Helsinki Declaration.

## 3. Results

### 3.1. Demographic and Clinical Characteristics

Most patients were middle-aged to older adults and male, with arteriovenous fistulas representing the predominant form of vascular access. The distribution of cases across the study years reflects a higher volume in 2020, with more even representation thereafter. Among nurses, diploma and bachelor’s qualifications and substantial clinical experience were common, and cannulation failures were overwhelmingly managed by re-needling by senior nurses, in line with unit policy for maintaining catheter use until repeated successful AVF cannulations are achieved. These patterns illustrate both a high-risk dialysis population and a strong reliance on experienced staff and protocol-driven cannulation management (Table 1).

### 3.2. Comorbidities

Diabetes mellitus (DM) was prevalent in 69.7% of the sample. Other notable comorbidities included ischemic heart disease (8.3%), peripheral vascular disease (9.2%), and recurrent stroke (6.1%), highlighting the multi-morbidity profile of the dialysis population (Table 2).

### 3.3. Cannulation Failure Incidence

**Figure 1:**
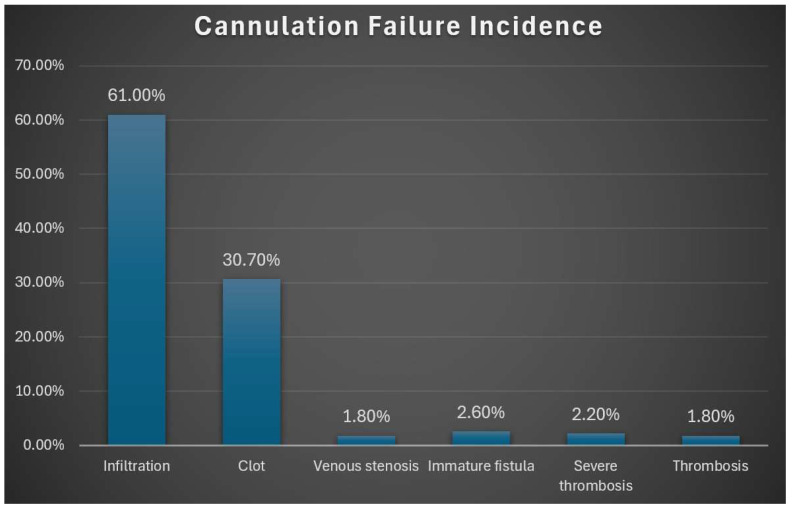
Distribution of Cannulation Failure Causes in Dialysis Patients (n = 228).

Infiltration emerged as the most common complication, accounting for 61.0% of cases of cannulation failure. Clot formation was the second-most frequent cause (30.7%), while immature fistulas (2.6%), severe thrombosis (2.2%), venous stenosis (1.8%), and thrombosis in general (1.8%) were less frequently observed. These results underscore the importance of identifying and managing infiltration early to reduce the risk of repeated failures and ensure continuity of dialysis (Figure 1).

### 3.4. Associations Between Cannulation Failure Incidence and Comorbidities

Chi-square analyses revealed no significant associations between cannulation failure types and common comorbidities such as hypertension and diabetes mellitus (*p* > 0.05). However, statistically significant associations were found with recurrent stroke (χ^2^ = 14.77, *p* = 0.011) and peripheral vascular disease (PVD) (χ^2^ = 19.02, *p* = 0.002). These findings suggest that neurological (i.e., Cerebral strokes) and vascular conditions (i.e., PVD) may contribute to an increased risk of cannulation-related complications, possibly due to impaired circulation or limb dysfunction (Table 3).

### 3.5. Influence of Patient and Nurse Demographics, Procedures, and Clinical Factors on Cannulation Failure Incidence

A highly significant association was observed between the type of cannulation failure and the action taken in response (χ^2^ = 456, *p* < 0.001). Infiltration and clot-related failures were consistently managed with re-needling, whereas cases involving immature fistulas and thrombosis were more often addressed by either starting with an existing catheter or discontinuing the session.

The year of cannulation failure occurrence was also significantly associated with the type of cannulation failure (χ^2^ = 110.01, *p* < 0.001), with infiltration peaking in 2020 and 2023, and clots being more prevalent in 2022 and 2024—possibly reflecting changes in practice patterns, staff rotation, or patient profiles over time.

In contrast, no statistically significant associations were found between cannulation failure types and age group (*p* = 0.228), nurse education (*p* = 0.555), nurse experience (*p* = 0.518), patient gender (*p* = 0.755), or type of vascular access (*p* = 0.305). Although these factors did not reach statistical significance, observed patterns—such as more venous stenosis in older patients and AVG users—may warrant further investigation in future studies (Table 4).

## 4. Discussion

Most patients in this cohort had hypertension and diabetes, reflecting a high overall comorbidity burden. Infiltration and clot formation were the predominant manifestations of cannulation failure. Cannulation failures occurred most frequently in patients with vascular comorbidities, with significant associations observed for recurrent stroke, thrombosis, and peripheral vascular disease, whereas no meaningful associations were found with nurse or patient demographic characteristics. Together, these findings suggest that underlying vascular pathology plays a more influential role in cannulation risk than staff experience or educational level in this setting.

Comorbidities may play a critical role in the high incidence of vascular access cannulation failure among nurses, as damaged vessels and poor circulation can hinder successful cannulation. These findings underscore the importance of specialized nursing skills and thorough assessment to minimize complications in this vulnerable population.

Previous studies found that diabetes and comorbidities significantly increase the risk of vascular access failure in hemodialysis patients. A meta-analysis by Yan et al. [21] revealed a statistically significantly higher rate of AVF failure in diabetic patients compared to non-diabetic patients (OR = 1.682; 95% CI, 1.429–1.981, *p* < 0.001). Wang [22] emphasized that for patients with ESRD, reliable vascular access is crucial for survival, and comorbidities can complicate treatment. Additionally, they note that during the first 6 months of new vascular access, a substantial number of cannulation-related complications occur, which can lead to increased morbidity.

Hill et al. [23] found that 24% of patients required surgical intervention to salvage poorly functioning AVFs before commencing hemodialysis, with 36% needing further surgical intervention after treatment began. Alsolami and Alobaidi [24] revealed that the knowledge of hemodialysis nurses about vascular access management was marginal, with only 53.3% demonstrating an adequate understanding. This knowledge gap, combined with patients’ complex health conditions, likely contributes to an increased risk of cannulation failure. While the sources confirm the challenges posed by comorbidities, they suggest that targeted nursing interventions and careful management can help mitigate these risks.

In this study, infiltration emerged as the most frequent complication among patients experiencing cannulation failure for hemodialysis, closely followed by clot formation. These findings indicate ongoing mechanical and procedural challenges in maintaining reliable vascular access, particularly when utilizing AVFs.

Infiltration during AV fistula cannulation refers to blood leaking from the vessel into the surrounding tissues when the needle has penetrated the vessel or is no longer fully within its lumen. It is usually linked to the patient’s movement or cannula dislocation or movement. With high blood flow and the cannula not well fixed, it may cause infiltration. Moreover, the quality of the vein may increase the risk of infiltration, as seen in cases of multiple venous stenoses. Infiltration most commonly occurs at the arterial or venous needle sites in the cannulated segment of the fistula, especially in areas with tortuous veins, recent cannulation sites, or segments with prior bruising or scarring [25]. Clinically, it presents sudden pain or burning at the needle site, swelling, tense or firm tissue around the access, bruising/hematoma, and often machine alarms from altered pressures or loss of adequate blood flow. Therapeutic measures include stopping the blood pump immediately, clamping and removing or repositioning the needle, applying gentle pressure, and then cold compresses (ice) in the acute phase to limit bleeding and hematoma, elevating the limb, and avoiding re-cannulation of the infiltrated area for several sessions [26].

Complications of arteriovenous fistulas may include venous hypertension with swelling (often due to central vein stenosis) or aneurysms and pseudoaneurysm formations of vascular access, usually identified by vascular surgeons, that pose patients at risk for certain complications, such as rupture, bleeding, or involvement of the overlying skin, with consequent cannulation difficulty [27]. Other complications of AVF also include thrombosis with access occlusion, outflow or anastomotic stenosis, access-site infection, steal syndrome with distal limb ischemia, high-output cardiac failure in large high-flow fistulas, and hematoma or significant post-cannulation bleeding [7].

The evidence suggests multiple potential cannulation complications exist. For example, a prior investigation into peripheral intravenous catheter failure revealed that among 11,830 catheters utilized by 8200 participants, a failure rate of 36% was observed. The incidence of occlusion/infiltration was 23%, phlebitis occurred in 12% of catheters, and dislodgement was reported in 7% of catheters [28].

Another study found that the most common complications in the cannulation sites were phlebitis, followed by infiltration, then extravasation [29]. Roşu et al. [7] found that thrombosis (30.8%) and hemorrhage (32.3%) were the most common early consequences. Diabetes and high blood pressure have a significant effect on these problems. They also found that thrombosis was the most common late consequence, and that people with high blood pressure were more likely to get it.

Al-Shameri et al. [30] reported thrombosis as the most frequent complication at 11.4%, followed by non-infectious fluid collections at 9.3%. Moreover, Behera et al. [31] found significant cannulation-related complications, with hematoma occurring in 83.7% of cases and thrombosis in 27.9%.

Several potential factors likely contribute to these complications, for example, repeated cannulation at the same sites can cause vessel trauma and increase the risk of infiltration and clot formation. Vessel fragility, common in chronic kidney disease patients due to coexisting conditions and prolonged vascular access use, further predisposes them to failed cannulation and complications. Patient movement during cannulation may lead to needle malposition and vascular injury, resulting in infiltration and even hematoma or thrombosis.

These results highlight the importance of enhanced nursing practice and training, underscoring the need for ongoing education, skill development, and adherence to best practices to improve patient outcomes and prolong vascular access longevity in the hemodialysis population.

The current study revealed that cannulation failure for hemodialysis has significant relationships with recurrent thrombosis and stroke and peripheral vascular disease, suggesting vascular impairments as substantial risk factors. No significant associations were observed with hypertension and diabetes, which may indicate that functional vascular changes, rather than metabolic control, have a greater impact on fistula stability.

This association of cannulation failure for hemodialysis with recurrent stroke and peripheral vascular disease highlights the importance of vascular health in achieving successful access, as recurrent strokes and peripheral vascular disease frequently involve compromised vessel integrity and reduced blood flow, both of which make cannulation procedures more challenging and increase the risk of failure [32].

Patients with a history of thrombosis and stroke frequently present with underlying vascular pathology, such as atherosclerosis or vessel fragility, further complicating vascular access maintenance [33]. Similarly, peripheral vascular disease, characterized by poor circulation and damaged vessel walls, can impede proper needle insertion and contribute to frequent complications and cannulation failure [34].

Umeno et al. [35] found that patients with higher small vessel disease scores had substantially increased risks of recurrent stroke, with hazard ratios rising from 1.34 to 2.21 per unit score increase. See et al. [36] identified that only 4% of hemodialysis patients had peripheral vascular disease, but those patients were at higher risk of AVF failure. Yap et al. [34] further demonstrated that early AVF failure was associated with increased overall mortality, suggesting interconnected vascular health risks.

These findings highlight the importance of thorough vascular assessment and tailored cannulation strategies for individuals with these comorbid conditions. For nursing practice, it is crucial to recognize such risk factors early, adapt techniques, and pursue advanced training to enhance successful cannulation and minimize adverse events with these high-risk patients.

The current findings revealed that cannulation failures were overwhelmingly managed by re-needling by senior nurses. However, fewer are managed by starting treatment with the existing catheter. When patients start dialysis emergently, a dialysis catheter is inserted and used until the AV fistula matures. Even after maturation, cannulation can fail; by hospital policy, the catheter is removed only after six successful AVF cannulations. If cannulation fails, dialysis is performed through the existing catheter for that session, the fistula is reassessed, and the catheter is retained until the fistula has been used effectively for several sessions. The policy of retaining the catheter until six successful AVF cannulations, and reverting to catheter use when cannulation fails, underscores the unit’s emphasis on staged transition from catheter to fistula, continuous reassessment of access maturity, and prevention of permanent fistula loss due to repeated traumatic attempts.

The current study found that the nursing intervention or cannulation failure management method (the action taken) was strongly associated with the type of cannulation failure, indicating that clinical responses are distinctly tailored to the specific complication. For example, interventions for infiltration may differ from those required for clot formation or vessel stenosis, demonstrating that nurses often employ distinctly tailored approaches depending on the situation at hand [31].

Takahashi et al. [37] demonstrated that care protocols including ultrasound pre-scanning and using flexible catheters could reduce failure rates from 29.2% to 11.1%—a relative risk reduction of 0.60. Santos-Costa et al. [38] further highlighted significant variations in nursing practices that may contribute to high complication rates, as observed in their oncology patient study (26%). These studies collectively suggest that targeted nursing interventions can substantially mitigate cannulation failure risks.

Additionally, the observed yearly variation in failure types suggests that practice patterns, procedural guidelines, and even patient or staff characteristics evolve over time. Shifts might reflect ongoing efforts to improve technique, changes in demographic factors such as age or comorbidity, or adjustments in staffing and training, all of which can influence the prevalence and type of cannulation failure encountered [31,39].

These results underscore the importance of flexibility and continuous learning in nursing practice. Proactive management strategies, ongoing education about best practices, and responsiveness to changing patient and procedural profiles are all key to optimizing cannulation outcomes and minimizing complications in the dynamic environment of hemodialysis care.

The study found no significant links between cannulation failure and nurse experience, education level, patient gender, or the type of vascular access used. This suggests that standardized procedural protocols across the unit are being followed, regardless of individual staff characteristics, effectively minimizing personal variability among nurses [40,41].

Multiple studies demonstrate that structured training can effectively normalize performance across different nurse backgrounds. Blick et al. [42] found that nurses’ first-attempt success rates for ultrasound-guided IV placement increased from 67% to 83% after standardized training, with performance becoming consistent across individuals. Spencer and Bardin-Spencer [43] similarly confirmed that a standardized curriculum improved procedural skills “regardless of level of experience” across interdisciplinary specialties.

However, the research also reveals significant current practice variations. Davis et al. [44] highlighted that only 47% of respondents had formal escalation procedures, and just two-thirds had standardized protocols, indicating substantial room for improvement in creating truly uniform clinical practices.

Such results highlight the strength of consistent, evidence-based guidelines and structured training in creating uniformity in practice. When protocols are clear and strictly implemented, factors such as experience or education may play a lesser role in daily outcomes, as nurses rely on shared approaches to cannulation rather than relying solely on personal judgment. Furthermore, this neutrality fosters an environment where equitable patient care is provided, with technical outcomes less dependent on individual nurse attributes and more on system-wide standards [40,45]. This underscores the importance of continual protocol updates and team-wide training to maintain high-quality, predictable hemodialysis care, ensuring all staff deliver the same level of safety and skill.

### Clinical Implications

Given that cannulation failure was strongly associated with vascular comorbidities such as recurrent thrombosis, stroke, and peripheral vascular disease, our findings support prioritizing early vascular assessment and close surveillance of high-risk patients, including more frequent Doppler evaluation and proactive referral to vascular surgery. The predominance of infiltration and clot formation as cannulation complications suggests a need to refine unit-level cannulation protocols, emphasizing vein-preservation strategies, careful needle positioning, and early detection of stenosis or thrombosis.

Because nurse experience and educational level were not significantly associated with cannulation failure, standardized training, checklists, and protocolized cannulation techniques may help maintain consistent outcomes across staff, even in units with variable experience levels. Integrating advanced risk assessment for vascular disease into routine patient evaluation and fostering a culture of learning are crucial steps toward lowering cannulation failure rates and enhancing patient outcomes. In settings with a high prevalence of diabetes and hypertension, integrating vascular access risk stratification into routine chronic disease management may help reduce access complications and preserve AV fistulas and grafts over time.

## 5. Conclusions

This study highlights the complexity of vascular access management in hemodialysis patients with a high burden of hypertension and diabetes mellitus. Cannulation failure was most often due to infiltration and clot formation and was strongly associated with vascular comorbidities, including recurrent thrombosis, stroke, and peripheral vascular disease, underscoring the central role of underlying vascular health in cannulation outcomes. The absence of significant associations with nursing experience or education suggests that standardized cannulation protocols may help achieve comparable outcomes across staff.

### 5.1. Recommendations

Dialysis centers are encouraged to establish and regularly review comprehensive cannulation protocols that are precisely tailored to the diverse risk profiles of their patient population, taking into account evolving best practices and evidence-based recommendations. It is vital to promote ongoing nurse education, focusing on advanced cannulation techniques and timely management of complications, while integrating hands-on skill development and regular competency assessments. Additionally, centers should implement vascular systematic evaluations for all patients—with special attention to those with a history of vascular disease or recurrent stroke—to proactively identify and address access challenges. Regular team meetings, audits, and surveys of cannulation practices are recommended to quickly recognize emerging trends or areas for improvement and foster a collaborative approach to enhancing patient safety and outcomes.

### 5.2. Limitations

The main limitations of this study include its retrospective cohort design, which may lead to incomplete or missing data, reliance on a single-center dataset that limits generalizability, and exclusion of detailed procedural variations or unmeasured confounding factors, such as operator skill and additional patient health parameters. Some potentially relevant factors were not included in the data collection sheet and may have influenced fistula cannulation, such as nutritional and inflammatory status and body mass index (BMI). We also acknowledge limitations in statistical design, such as the absence of multivariable analysis (e.g., logistic regression) and the small cell sizes.

## Figures and Tables

**Table 1:** Demographic and Clinical Characteristics of Dialysis Patients and Attending Nurses (n = 228).

Variables		n	%
Patient age	<=40	34	14.90%
	41–60	97	42.50%
	61–80	82	36.00%
	>=80	15	6.60%
Patient age (Mean ± SD)	57.02 ± 8.23		
Patient gender	Male	155	68.00%
	Female	73	32.00%
Year	2020	93	40.80%
	2021	13	5.70%
	2022	51	22.40%
	2023	46	20.20%
	2024	25	11.00%
Nurse education	Diploma in Nursing	128	56.10%
	Bachelor’s degree	100	43.90%
Nurse experience	<1	8	3.50%
	1–5	69	30.30%
	>5	151	66.20%
Action	Re-needling by a senior nurse	213	93.40%
	Starting with the existing catheter	10	4.40%
	Discontinue treatment	5	2.20%
Dialysis Access	AV-Fistula	197	86.40%
	AV-Graft	31	13.60%

Abbreviations: SD, standard deviation; AV, arteriovenous; AV-fistula, arteriovenous fistula; AV-graft, arteriovenous graft.

**Table 2:** Prevalence of Comorbidities among Dialysis Patients (n = 228).

Comorbidities	n	%
Peripheral Vascular Disease	21	9.2%
Recurrent Stroke	14	6.1%
Ischemic Heart Disease	19	8.3%
DM	159	69.7%

Abbreviation: DM, diabetes mellitus.

**Table 3:** Association between Cannulation Failure Types and Patient Comorbidities.

Association		Cannulation Failure Incidence												Chi-Square	*p*
		Infiltration		Clot		Venous Stenosis		Immature Fistula		Severe Thrombosis		Thrombosis			
		n	%	n	%	n	%	n	%	n	%	n	%		
HTN	other	0	0.0%	0	0.0%	0	0.0%	0	0.0%	0	0.0%	0	0.0%	0.91	0.969
	HTN	139	61.0%	70	30.7%	4	1.8%	6	2.6%	5	2.2%	4	1.8%		
DM	other	37	16.2%	26	11.4%	2	0.9%	1	0.4%	2	0.9%	1	0.4%	3.98	0.551
	DM	102	44.7%	44	19.3%	2	0.9%	5	2.2%	3	1.3%	3	1.3%		
Ischemic Heart Disease	other	126	55.3%	66	28.9%	4	1.8%	4	1.8%	5	2.2%	4	1.8%	6.9	0.228
	Ischemic Heart Disease	13	5.7%	4	1.8%	0	0.0%	2	0.9%	0	0.0%	0	0.0%		
Recurrent Stroke	other	130	57.0%	67	29.4%	2	0.9%	6	2.6%	5	2.2%	4	1.8%	14.77	0.011
	Recurrent Stroke	9	3.9%	3	1.3%	2	0.9%	0	0.0%	0	0.0%	0	0.0%		
Peripheral Vascular Disease	other	134	58.8%	59	25.9%	2	0.9%	5	2.2%	4	1.8%	3	1.3%	19.02	0.002
	Peripheral Vascular Disease	5	2.2%	11	4.8%	2	0.9%	1	0.4%	1	0.4%	1	0.4%		

Abbreviations: HTN, Hypertension; DM, Diabetes mellitus.

**Table 4:** Association of cannulation failure incidence with patient and nurse demographics, procedures, and patient clinical data.

Association		Cannulation Failure Incidence												Chi-Square	*p*
		Infiltration		Clot		Venous Stenosis		Immature Fistula		Severe Thrombosis		Thrombosis			
		n	%	n	%	n	%	n	%	n	%	n	%		
Age	<=40	16	11.5%	15	21.4%	1	25.0%	1	16.7%	1	20.0%	0	0.0%	18.7	0.228
	41–60	69	49.6%	23	32.9%	0	0.0%	1	16.7%	1	20.0%	3	75.0%		
	61–80	43	30.9%	29	41.4%	3	75.0%	3	50.0%	3	60.0%	1	25.0%		
	>=80	11	7.9%	3	4.3%	0	0.0%	1	16.7%	0	0.0%	0	0.0%		
Nurse education	Diploma in Nursing	83	59.7%	33	47.1%	3	75.0%	4	66.7%	3	60.0%	2	50.0%	3.96	0.555
	Bachelor’s degree	56	40.3%	37	52.9%	1	25.0%	2	33.3%	2	40.0%	2	50.0%		
Nurse experience	<1	4	2.9%	4	5.7%	0	0.0%	0	0.0%	0	0.0%	0	0.0%	9.15	0.518
	1–5	41	29.5%	25	35.7%	1	25.0%	0	0.0%	0	0.0%	2	50.0%		
	>5	94	67.6%	41	58.6%	3	75.0%	6	100.0%	5	100.0%	2	50.0%		
Action	Re-needling by a senior nurse	139	100.0%	70	100.0%	4	100.0%	0	0.0%	0	0.0%	0	0.0%	456	<0.001
	Starting with the existing catheter	0	0.0%	0	0.0%	0	0.0%	6	100.0%	0	0.0%	4	100.0%		
	Discontinue treatment	0	0.0%	0	0.0%	0	0.0%	0	0.0%	5	100.0%	0	0.0%		
Year	2020	72	51.8%	17	24.3%	2	50.0%	2	33.3%	0	0.0%	0	0.0%	110.01	<0.001
	2021	1	0.7%	9	12.9%	1	25.0%	0	0.0%	2	40.0%	0	0.0%		
	2022	18	12.9%	27	38.6%	0	0.0%	2	33.3%	2	40.0%	2	50.0%		
	2023	45	32.4%	1	1.4%	0	0.0%	0	0.0%	0	0.0%	0	0.0%		
	2024	3	2.2%	16	22.9%	1	25.0%	2	33.3%	1	20.0%	2	50.0%		
Patient gender	Male	96	69.1%	45	64.3%	4	100.0%	4	66.7%	3	60.0%	3	75.0%	2.63	0.755
	Female	43	30.9%	25	35.7%	0	0.0%	2	33.3%	2	40.0%	1	25.0%		
Dialysis Access	AV-Fistula	120	86.3%	61	87.1%	2	50.0%	5	83.3%	5	100.0%	4	100.0%	6.01	0.305
	AV-Graft	19	13.7%	9	12.9%	2	50.0%	1	16.7%	0	0.0%	0	0.0%		

Abbreviations: AV, arteriovenous; AV-fistula, arteriovenous fistula; AV-graft, arteriovenous graft.

## Data Availability

The data presented in this study are available on request from the corresponding author. The data are not publicly available due to privacy and ethical restrictions.

## Abbreviations

The following abbreviations are used in this manuscript:

AVF	Arteriovenous fistula
AVG	Arteriovenous graft
ESRD	End-Stage Renal Disease
KFMMC	King Fahad Military Medical Complex
VA	Vascular Access

## References

[1] Smith K., Ayars C. (2025). Improving vascular access knowledge and assessment skill of hemodialysis staff. J. Osteopath. Med..

[2] Battistella A., Linger M., Nguyen A.T., Madukwe D., Roy-Chaudhury P., Tan W. (2024). Rebuilding vascular access: From the viewpoint of mechanics and materials. Front. Bioeng. Biotechnol..

[3] Lok C.E., Huber T.S., Lee T., Shenoy S., Yevzlin A.S., Abreo K., Allon M., Asif A., Astor B.C., Glickman M.H. (2020). KDOQI Clinical Practice Guideline for Vascular Access: 2019 Update. Am. J. Kidney Dis..

[4] Ștefan G., Podgoreanu E., Mircescu G. (2025). Patterns and outcomes of vascular access in hemodialysis: A nationwide registry-based study from Romania. Ren. Fail..

[5] Venegas-Ramírez J., Hernández-Fuentes G.A., Palomares C.S., Diaz-Martinez J., Navarro-Cuellar J.I., Calvo-Soto P., Duran C., Tapia-Vargas R., Espíritu-Mojarro A.C., Figueroa-Gutiérrez A. (2025). Vascular Access Type and Survival Outcomes in Hemodialysis Patients: A Seven-Year Cohort Study. Medicina.

[6] Lawson J.H., Niklason L.E., Roy-Chaudhury P. (2020). Challenges and novel therapies for vascular access in haemodialysis. Nat. Rev. Nephrol..

[7] Roşu C.D., Bolintineanu S.L., Căpăstraru B.F., Iacob R., Stoicescu E.R., Petrea C.E. (2025). Risk Factor Analysis in Vascular Access Complications for Hemodialysis Patients. Diagnostics.

[8] Peters C., Menkiti I., Desalu I., Thomas M. (2013). Multiple venous thrombosis complicating central venous cannulation in a non cancer patient—A case report. J. West. Afr. Coll. Surg..

[9] Coventry L.L., Hosking J.M., Chan D.T., Coral E., Lim W.H., Towell-Barnard A., Twigg D.E., Rickard C.M. (2019). Variables associated with successful vascular access cannulation in hemodialysis patients: A prospective cohort study. BMC Nephrol..

[10] Alshehri M.A., Alkhlady H.Y., Awan Z.A., Algethami M.R., Al Mahdi H.B., Daghistani H., Orayj K. (2025). Prevalence of chronic kidney disease in Saudi Arabia: An epidemiological population-based study. BMC Nephrol..

[11] Abu El-Kass S., Ahmed N., Kannan T., Abu Shediq N., El Dirani E. (2024). Nurses’ knowledge toward hemodialysis vascular access devices: A cross-sectional study in Palestine. Sage Open Med..

[12] Parisotto M.T., Pelliccia F., Grassmann A., Marcelli D. (2017). Elements of dialysis nursing practice associated with successful cannulation: Result of an international survey. J. Vasc. Access.

[13] Stolic R.V., Trajkovic G.Z., Kostic M.M., Lazic B.D., Odalovic B., Smilic T.N., Mitic J.S. (2017). Cannulation Technique and Arteriovenous Fistula Survival in Older Adult Patients on Hemodialysis. Nephrol. Nurs. J..

[14] Hod T., Desilva R.N., Patibandla B.K., Vin Y., Brown R.S., Goldfarb-Rumyantzev A.S. (2014). Factors predicting failure of AV “fistula first” policy in the elderly. Hemodial. Int..

[15] Parisotto M.T., Schoder V.U., Miriunis C., Grassmann A.H., Scatizzi L.P., Kaufmann P., Stopper A., Marcelli D. (2014). Cannulation technique influences arteriovenous fistula and graft survival. Kidney Int..

[16] Wilmink T., Hollingworth L., Stevenson T., Powers S. (2017). Is early cannulation of an arteriovenous fistula associated with early failure of the fistula?. J. Vasc. Access.

[17] Ghonemy T.A., Farag S.E., Soliman S.A., Amin E.M., Zidan A.A. (2016). Vascular access complications and risk factors in hemodialysis patients: A single center study. Alex. J. Med..

[18] Abdosh K., Suliman I., Ahmed G. (2020). Complications of permanent vascular access in hemodialysis patients. J. Egypt. Soc. Nephrol. Transplant..

[19] Aljuaid M.S., Aseeri A.A., Alzahrani A.M., Alomari I.A.H., Nefaie A.T.M.A., Alotibai A.F., Al Gthami M.M., Ali L.A., Alharthi S.E.R., Alshmrani A.S.A. (2024). Vascular Access Complications In Hemodialysis: Comprehensive Review Of Early And Late Manifestations, Risk Factors, And Clinical Management Strategies. Rev. Diabet. Stud..

[20] Harwood L., Wilson B., Goodman M. (2017). Cannulation Outcomes of the Arteriovenous Fistula for Hemodialysis: A Scoping Review. Nephrol. Nurs. J..

[21] Yan Y., Ye D., Yang L., Ye W., Zhan D., Zhang L., Xiao J., Zeng Y., Chen Q. (2018). A meta-analysis of the association between diabetic patients and AVF failure in dialysis. Ren. Fail..

[22] Wang J. (2024). Comprehensive Nursing Care for Vascular Access in Hemodialysis Patients with End-Stage Renal Disease. Int. J. Public Health Med. Res..

[23] Hill K., Xu Q., Jaensch A., Esterman A., Le Leu R., Childs J., Juneja R., Jesudason S. (2021). Outcomes of arteriovenous fistulae cannulation in the first 6 weeks of use: A retrospective multicenter observational study. J. Vasc. Access.

[24] Alsolami E., Alobaidi S. (2024). Hemodialysis nurses’ knowledge, attitude, and practices in managing vascular access: A cross-sectional study in Saudi Arabia. Medicine.

[25] Parisotto M., Pancirova J. (2018). Vascular Access Cannulation and Care: A Nursing Best Practice Guide for Arteriovenous Fistula.

[26] Fowler M., Lozano A.M., Krause J., Bednarz P., Pandey S., Ghayour M., Zhang Q., Veiseh O. (2025). Guiding vascular infiltration through architected GelMA/PEGDA hydrogels: An in vivo study of channel diameter, length, and complexity. Biomater. Sci..

[27] Al-Jaishi A.A., Liu A.R., Lok C.E., Zhang J.C., Moist L.M. (2017). Complications of the Arteriovenous Fistula: A Systematic Review. J. Am. Soc. Nephrol..

[28] Marsh N., Larsen E.N., Takashima M., Kleidon T., Keogh S., Ullman A.J., Mihala G., Chopra V., Rickard C.M. (2021). Peripheral intravenous catheter failure: A secondary analysis of risks from 11,830 catheters. Int. J. Nurs. Stud..

[29] Kaphan K., Auypornsakul S., Somno J., Wongwattananan W., Jamsittikul K., Baicha W., Somsri S., Sawatrak T. (2024). The Prevalence and Associated Factors of Peripheral Intravenous Complications in a Thai Hospital. J. Infus. Nurs..

[30] Al-Shameri I., KhudaBux G., Al-Ganadi A. (2021). Prospective evaluation of factors associated with arteriovenous fistula primary failure and complications in hemodialysis patients: A single center-study. Cardiol. Vasc. Res..

[31] Behera M.R., John E.E., Thomas A., David V.G., Alexander S., Mohapatra A., Valson A.T., Jacob S., Kakde S., Koshy P.M. (2022). Difficult cannulation of hemodialysis arteriovenous fistula—Role of imaging in access management (Dicaf Study). J. Vasc. Access.

[32] Mark P.B., Jhund P.S., Walters M.R., Petrie M.C., Power A., White C., Robertson M., Connolly E., Anker S.D., Bhandari S. (2021). Stroke in Hemodialysis Patients Randomized to Different Intravenous Iron Strategies: A Prespecified Analysis from the PIVOTAL Trial. Kidney360.

[33] Boehme A.K., Esenwa C., Elkind M.S.V. (2017). Stroke Risk Factors, Genetics, and Prevention. Circ. Res..

[34] Yap Y.-S., Chi W.-C., Lin C.-H., Liu Y.-C., Wu Y.-W. (2021). Association of early failure of arteriovenous fistula with mortality in hemodialysis patients. Sci. Rep..

[35] Umeno T., Yamashita A., Mizota T., Uramatsu T., Matsuo T. (2022). Predictive Value of Total Small-Vessel Disease Score for Recurrent Stroke in Patients Undergoing Maintenance Hemodialysis. J. Stroke Cerebrovasc. Dis..

[36] See Y.P., Cho Y., Pascoe E.M., Cass A., Irish A., Voss D., Polkinghorne K.R., Hooi L.S., Ong L.M., Paul-Brent P.A. (2020). Predictors of Arteriovenous Fistula Failure: A Post Hoc Analysis of the Favoured Study. Kidney360.

[37] Takahashi T., Murayama R., Abe-Doi M., Miyahara-Kaneko M., Kanno C., Nakamura M., Mizuno M., Komiyama C., Sanada H. (2020). Preventing peripheral intravenous catheter failure by reducing mechanical irritation. Sci. Rep..

[38] Santos-Costa P., Paiva-Santos F., Sousa L.B., Bernardes R.A., Ventura F., Fearnley W.D., Salgueiro-Oliveira A., Parreira P., Vieira M., Graveto J. (2022). Nurses’ Practices in the Peripheral Intravenous Catheterization of Adult Oncology Patients: A Mix-Method Study. J. Pers. Med..

[39] Arvind Raut A., Uddanwadikar R., Padole P., Upadhye S.V., Fiske G.A., Bhadane B.K. (2025). Arteriovenous Fistula Failure in Hemodialysis: Mechanisms, Risk Factors, and Management Strategies. J. Neonatal Surg..

[40] Hosney M.S., Mohammed I.R., Mohammed H.E. (2022). Effect of Nursing Guidelines for Buttonhole Cannulation Technique of Arteriovenous Fistula on Reducing its Complications among Hemodialysis Patients. Minia Sci. Nurs. J..

[41] Lee Y.-N., Kim E.Y. (2022). Experiences of Nurses Caring for Hemodialysis Patients: A Qualitative Meta-Synthesis Study. Korean J. Adult Nurs..

[42] Blick C., Vinograd A., Chung J., Nguyen E., Abbadessa M.K.F., Gaines S., Chen A. (2021). Procedural competency for ultrasound-guided peripheral intravenous catheter insertion for nurses in a pediatric emergency department. J. Vasc. Access.

[43] Spencer T.R., Bardin-Spencer A.J. (2020). Pre- and post-review of a standardized ultrasound-guided central venous catheterization curriculum evaluating procedural skills acquisition and clinician confidence. J. Vasc. Access.

[44] Davis M.B.H., Takashima M., Girgenti C., Ullman A.J. (2020). An international survey of pediatric and neonatal clinicians’ vascular access practice: PediSIG assessment of vascular access, education, and support (PAVES) catheter selection. Br. J. Nurs..

[45] Liu Z., Bible J., Petersen L., Roy-Chaudhury P., Geissler J., Brouwer-Maier D., Singapogu R. (2021). Measuring Cannulation Skills for Hemodialysis: Objective Versus Subjective Assessment. Front. Med..

